# Non-extraction orthodontic treatment for severe dental crowding using miniscrew-assisted rapid maxillary expansion

**DOI:** 10.1093/jscr/rjac509

**Published:** 2022-11-14

**Authors:** Sara Altamash, Hasnain Sakrani, Naseer Ahmed, Anand Marya, Artak Heboyan

**Affiliations:** Department of Orthodontics, Altamash Institute of Dental Medicine, Karachi, Pakistan; Department of Orthodontics, Altamash Institute of Dental Medicine, Karachi, Pakistan; Department of Prosthodontics, Altamash Institute of Dental Medicine, Karachi, Pakistan; Department of Orthodontics, Faculty of Dentistry, University of Puthisastra, Phnom Penh, Cambodia; Center of Transdisciplinary Research, Saveetha Dental College, Saveetha Institute of Medical and Technical Science, Saveetha University, Chennai, India; Department of Prosthodontics, Faculty of Stomatology, Yerevan State Medical University after Mkhitar Heratsi, Yerevan, Armenia

## Abstract

Malocclusion is caused by an incorrect relationship between the teeth and jaws, which leads to an abnormal variation of normal occlusion. Crowding is an increasingly common type of malocclusion caused by a discrepancy in tooth-jaw size that leads to twisted and misaligned teeth. Two methods to treat this malalignment are tooth material reduction and arch width expansion. Of these two treatment options, the latter is preferred because it enables orthodontists to avoid extractions and increase the dental arch perimeter by widening the jaws; this resolves crowding and accommodates existing teeth. This case report describes a unique approach in the treatment of a patient with severe crowding by an orthopaedic widening of the dental arches using a skeletally anchored rapid palatal expander. Under favorable circumstances, this approach can serve as a robust method to treat patients with severe crowding and may be used as an alternative to extraction.

## INTRODUCTION

Orthodontic treatment aims to achieve facial equilibrium and a balanced relationship [1] between teeth and underlying skeletal structures for all malocclusions, especially in patients with dental crowding [2]. Rapid palatal expansion is becoming a popular treatment option for all patients with crowding because it allows predictable enhancement of dental arch perimeter [3] rather than reduction of tooth mass [4]. Miniscrew-assisted rapid maxillary expansion [5] is an increasingly popular method for stable [6] orthopedic expansion in arch width, especially in growing patients. Here, we describe an uncommon orthodontic approach to treating a patient with severe crowding; instead of dental extraction, treatment comprised increasing arch width and perimeter, enhanced by using a state-of-the-art miniscrew-assisted rapid palatal expander.

## CASE HISTORY

**Figure 1:**
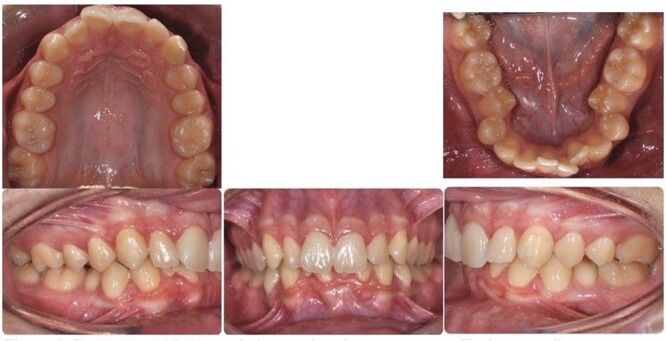
Pretreatment intra-oral photos showing severe mandibular crowding.

**Figure 2:**
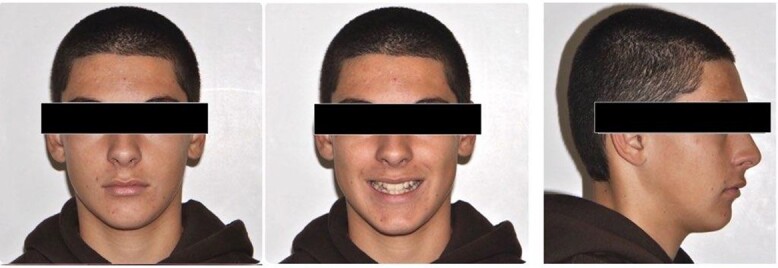
Pretreatment extra-oral photos.

A 15-year-old male patient of Asian origin presented for orthodontic treatment with the chief complaint of crowding in his lower front teeth. Intra-oral clinical examination revealed severe crowding in the mandible, mild crowding in the maxilla, a bilateral class II canine relationship, retroclined upper incisors, impinging deep overbite and overjet of 5 mm (Fig. 1). His convex soft tissue profile and smile analysis revealed a decreased incisal show (Fig. 2).

**Figure 3:**
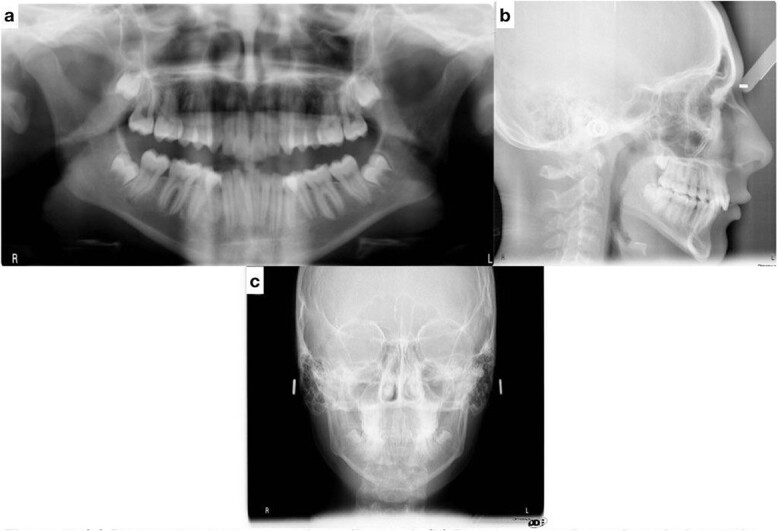
(**a**) Pretreatment panoramic radiograph; (**b**) pretreatment lateral cephalometric radiograph; (**c**) pretreatment posterior-anterior cephalometric radiograph.

Lateral cephalometric radiograph analysis showed a mild mandibular deficiency and high mandibular plane angle, retroclined maxillary incisors and proclined mandibular incisors (Fig. 3b). Panoramic radiography showed impacted third molars; rocky mountain analysis on the posterior-anterior cephalometric radiograph showed a narrow maxilla in the transverse dimension (Fig. 3c).

Skeletal treatment objectives included orthopedic expansion of the maxilla in the transverse dimension. Dental treatment aimed to increase intermolar width, upright the mandibular posterior segments buccolingually, proclining the maxillary incisors, achieve a class I canine relationship and position the lower incisors optimally to achieve ideal overjet and overbite.

**Figure 4:**
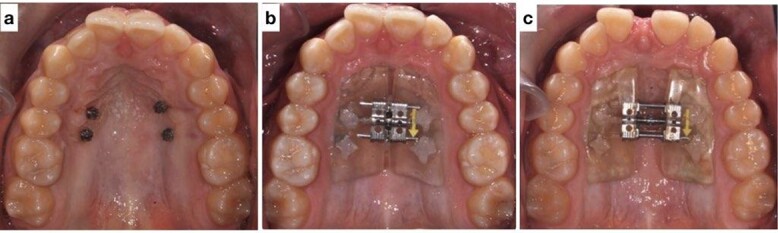
(**a**) Mini screws placed for the skeletally anchored rapid palatal expander. (**b**) Pre-expansion photo of the skeletally anchored rapid palatal expander; (**c**) post-expansion photo of the skeletally anchored rapid palatal expander.

Treatment was initiated using a miniscrew-assisted rapid palatal expander to widen the upper arch by 4 mm (Fig. 4). The patient cooperated with maintaining oral hygiene, and the parents assisted with turning the expander. After expansion, the molars were banded, and the remaining teeth were bonded with 0.022" × 0.028" slot standard edgewise bands and Mclaughlin, Bennett and Trevisi (MBT) prescription brackets. Initially, both arches were leveled and aligned through thermo-activated NiTi wires: 0.012" NiTi, 0.016" NiTi and 0.017" × 0.025" Nitinol wires. Progress X-rays and repositioning of bands and brackets were performed as needed to ensure root parallelism. Arch coordination was then attained with upper and lower 0.019" × 0.025" Stainless steel (SS) wires. These wires also leveled the Curve of Spee, uprighted the posterior teeth buccolingually, intruded the lower anterior teeth and coordinated the arches. Residual spaces were closed with power chains, and detailed bends were made. The patient was debonded after finishing and detailing, and retainers were delivered. The retainers provided were upper removable wrap-around Hawley and lower fixed lingual retainers.

**Figure 5:**
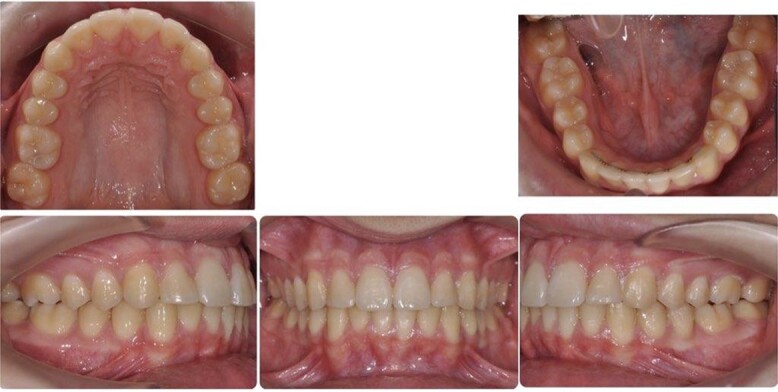
Post-treatment intra-oral photos showing severe mandibular crowding

**Figure 6:**
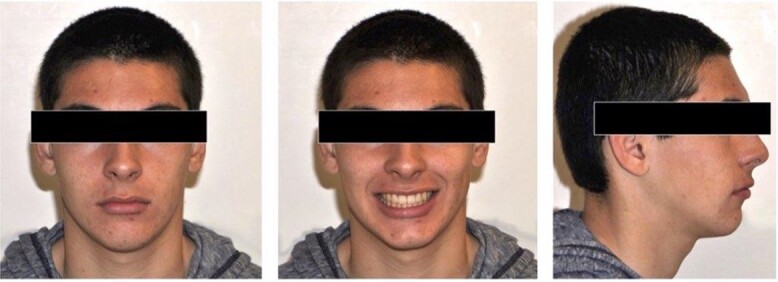
Post-treatment extra-oral photos

**Figure 7:**
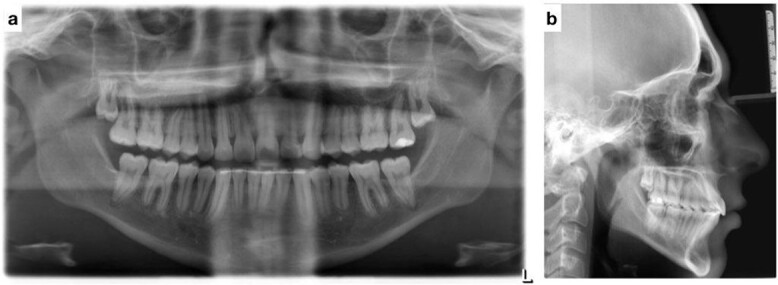
(**a**) Post-treatment panoramic radiograph; (**b**) post-treatment lateral cephalometric radiograph

Post-treatment clinical examination showed that crowding was relieved by a 4-mm increase in the intermolar width of the maxillary and mandibular arches. In addition, a class I molar and canine relationship was achieved with good occlusion (Fig. 5) and a pleasing smile (Fig. 6). Post-treatment lateral cephalometric radiographs showed that the mandible had grown forward in the sagittal dimension, improving the skeletal profile (Fig. 7b). The upper incisors were proclined by 15° to achieve ideal labio-lingual inclination for optimum aesthetics; the lower incisor was optimally positioned to achieve ideal overjet and overbite (Fig. 7b). Mandibular anterior teeth were intruded to correct the impinging overbite and level the Curve of Spee. Finally, the patient was referred to an oral surgeon for extraction of the third molars.

## DISCUSSION

Orthodontists must gain a greater awareness that crowding is an increasingly common type of malocclusion [2] caused by abnormalities in the dentition, jaws, or both [1]. The severity of this tooth size-arch length discrepancy is typically measured by visual examination methods and classified as mild, moderate or severe crowding [7].

Dental [7] and skeletal [8] measurements reveal that crowding is caused by excess tooth size, decreased arch width, or a combination of large teeth and narrow jaws. Three well-known treatment options for this type of malalignment in adolescent patients are interproximal reduction [9], extractions [4], and expansion [2]. Interproximal reduction is typically only used to treat patients with mild-to-moderate crowding, whereas extraction and expansion are standard treatment options for patients with all types of crowding. In patients with standard arch width and excess tooth size, extractions are recommended, especially for those with severe mandibular crowding (>6 mm) [4]. In contrast, in the presence of a normal-sized dentition and decreased arch width, expansion is the treatment of choice because it enables widening of the dental arches, which predictably increases the arch perimeter [3] and provides a given amount of transverse expansion and accommodates existing teeth. Predicting this relationship helps promote rapid palatal expanders in patients with crowding; these expanders facilitate non-extraction orthodontic treatment, which is the treatment preference for most modern orthodontists [10–12]. In addition, to achieve more significant orthopedic skeletal change, especially in a growing patient, a miniscrew-assisted rapid maxillary expander [5, 6] can be used in patients with a true skeletal transverse discrepancy [8], rather than a tooth tissue-borne rapid palatal expander. Subsequent widening of the dental arches from this treatment protocol is generally considered stable [6], as it exhibits minimum relapse, especially in patients with fixed prolonged retention.

In this case report, severe mandibular crowding was caused by a decreased arch length because of a narrow maxilla (transverse skeletal discrepancy) that was diagnosed via posterior-anterior cephalometric radiography: the skeletal width of the maxilla and mandible was measured based on skeletal landmarks and norms, as developed by Ricketts [8]. This decrease in maxillary arch width also restricted the mandibular arch width, resulting in severe mandibular crowding and collapsed mandibular posterior segments. Treatment was initiated with the expansion of the maxillary arch, thereby widening dental arches and creating an increased arch perimeter; this unraveled the crowding and allowed uprighting of the collapsed mandibular buccal segments with routine orthodontic treatment. The key to the alleviation of crowding in this patient was increasing the transverse dimension by using a miniscrew-assisted rapid maxillary expander [5, 6], rather than a tooth tissue-borne rapid palatal expander, which enabled a 4-mm increase in the width of the maxillary dental arch to be achieved by solid orthopedic expansion. The success of this maxillary expansion increased the mandibular intermolar width by 4 mm, which created sufficient space to relieve severe crowding without extraction. In order to ensure the post-treatment long-term stability of these widened arches, the retention protocol included a combination of fixed and removable prolonged retention procedures. Therefore, this case report highlights the importance of increasing the arch width and arch perimeter [3] to alleviate dental crowding during orthodontic treatment, thereby avoiding extractions and facilitating the maximum preservation of dental units.

## CONCLUSION

In the treatment of patients with severe dental crowding, orthodontists should critically examine and diagnose the biological basis of the crowding and address the true cause of the malocclusion; moreover, as an alternative to orthodontic extractions, orthodontists should consider treatment techniques that focus on widening dental arch dimensions with miniscrew-assisted rapid palatal expanders.

## AUTHORS’ CONTRIBUTIONS

SA, HS and NA were involved in collection of records; SA, AM and AH took the responsibility of analysis and treatment plan; SA, HS and NA did treatment provision; SA, HS, AM and AH led the drafting of the manuscript, editing and revisions. All authors agreed to the final version of this manuscript for submission.

## CONSENT

Informed consent was acquired from the patient for the use of his images and treatment data for research and publication purposes.

## DATA AVAILABILITY

The authors can provide data related to this manuscript on reasonable request.

## CONFLICT OF INTEREST STATEMENT

No funding was acquired or applied for the purpose of this manuscript.

## FUNDING

None.
